# Multi-Image Compression–Encryption Algorithm Based on Compressed Sensing and Optical Encryption

**DOI:** 10.3390/e24060784

**Published:** 2022-06-02

**Authors:** Jingjin Wei, Miao Zhang, Xiaojun Tong

**Affiliations:** Computer Science and Technology, Harbin Institute of Technology, Weihai 264200, China; 20s030163@stu.hit.edu.cn (J.W.); tong_xiaojun@hit.edu.cn (X.T.)

**Keywords:** compressed sensing, fractional-order chaotic system, double random phase encoding, image compression

## Abstract

In order to achieve large-capacity, fast and secure image transmission, a multi-image compression–encryption algorithm based on two-dimensional compressed sensing (2D CS) and optical encryption is proposed in this paper. Firstly, the paper uses compressed sensing to compress and encrypt multiple images simultaneously, and design a new structured measurement matrix. Subsequently, double random phase encoding based on the multi-parameter fractional quaternion Fourier transform is used to encrypt the multiple images for secondary encryption, which improves the security performance of the images. Moreover, a fractional-order chaotic system with more complex chaotic behavior is constructed for image compression and encryption. Experimental results show that the algorithm has strong robustness and security.

## 1. Introduction

As part of the advances in networking and communication technology, extensive research has been conducted on how to transmit images in real-time and securely. In recent years, chaos has been widely used in data transmission and image encryption due to its inherent characteristics, such as sensitivity to initial conditions and inherent randomness. In chaotic mapping, the fractional-order chaotic system, as a generalization of integer-order chaos, has more complex dynamics than the integer-order chaotic system, and thus has more abundant application value in the field of image encryption. For example, Ding et al. [1] described an image encryption scheme using two chaotic systems, including a fractional-order Henon chaotic mapping and a four-dimensional hyperchaotic system. Xu et al. [2] proposed a fractional-order chaotic system based on the Hopfield neural network as a pseudo-random number generator, and constructed a new image encryption algorithm with the multiple hash index chain. The fractional chaotic system in the above paper is mainly applied to image encryption, but it can also be applied to the two processes of image compression and encryption concurrently. Hu et al. [3] used a fractional-order simplest memristive chaotic system and compressive sensing to compress and encrypt images. However, they did not consider applying the fractional-order chaotic system to the image compression part.

While ensuring image security, to save more transmission bandwidth, transmission time and storage space, compressed sensing (CS) has gradually been applied to image compression and image encryption. CS theory [4] means that under the condition of a far smaller than Nyquist sampling rate, discrete signal samples under random sampling can be nonlinearly reconstructed into original signals, but they need to rely on the two preconditions of sparsity and incoherence. Since CS was proposed, many compression and encryption algorithms based on CS have appeared. Belyaev et al. [5] studied an iterative threshold-based compressed sensing video restoration algorithm. Huang et al. [6] embedded the encrypted image into the carrier image after SHA-3 and CS compression to achieve multi-image visual security. Gan et al. [7] fully combined information entropy and CS for color image compression and encryption. Ye et al. [8] proposed an image compression and encryption algorithm based on the elliptic curve and CS. At present, most of the objects of compression–encryption algorithms are single color images and multiple grayscale images, and how to compress and encrypt multiple color images is also a situation worthy of research.

Optical encryption technology has been widely used in the field of multi-encryption image encryption because of its powerful computing capability, multi-dimensional storage and parallel processing capability. The double random phase encoding [9] with a simple implementation process and high robustness is a very classical optical encryption system, but it cannot resist attacks such as selected plaintext attacks and known-plaintext attacks. Therefore, to improve the security of the encryption system, the Fourier transform domain used in double random phase encoding technology has been extended to other transform fields, such as the fractional Fourier transform and Gyrator transform domain. In addition, the combination of the optical encryption system, chaos and the CS system can also enhance the performance of image compression–encryption systems. Sun et al. [10] presented a multi-image encryption algorithm based on multi-dimensional chaos and cascade rotator transformation. Huo et al. [11] adopted CS and orthogonal coding to carry out data sampling and the integration of multiple images, proposing an encryption algorithm combining chaos matrix and double random phase encoding. However, the current multi-color image compression and encryption method combining 2D CS and the optical encryption system has been considered less.

Based on the above discussion, to reduce the amount of transmitted data and improve the encryption capacity and security, a multi-image compression–encryption algorithm based on 2D CS and optical encryption technology is proposed, which is suitable for color images and grayscale images. The innovative design of the overall idea of this paper is as follows. The application of quaternion can improve the encryption capability without increasing the complexity of the encryption system, but currently, few algorithms apply it to image compression and encryption. When double random phase encoding technology is applied to image encryption, two parts of data, the real part and the imaginary part, are generated, which means the transmission of the encrypted image data needs to pass twice the data. Similarly, the application of double random phase encoding based on a multi-parameter fractional quaternion Fourier transform (MPFrQFT) to encrypted data can also result in an exponential increase in data volume [12]. However, when multiple images are combined with double random phase encoding based on an MPFrQFT transform for image encryption, the amount of data needed to be transmitted is the same as the original image data. Meanwhile, when image compression and multi-image encryption are combined, the amount of data to be transmitted can be greatly reduced, and the secure and efficient compression and encryption effect can be achieved.

This paper firstly designed a new fractional-order chaotic map, and the generated chaotic sequences were applied to the whole algorithm to improve its security of this algorithm. Secondly, a new deterministic random measurement matrix was constructed for 2D CS, which reduces the complexity of constructing the measurement matrix and makes the image easy to reconstruct. Thirdly, an improved image encryption algorithm was designed. The two-dimensional Joseph scrambling algorithm can confuse the row and column places of all global pixels simultaneously, while the diffusion algorithm can compress and encrypt in the same process, protecting the image information while reducing the data. Fourthly, double random phase coding based on MPFrQFT is used to encrypt multiple images. The algorithm can compress and encrypt multiple images, which improves the encryption capacity and image security.

The rest of this paper is organized as follows. Section 2 introduces some preliminary work. Section 3 gives a description of the proposed multi-image compression and encryption algorithm. Section 4 gives the experimental results and analysis of the algorithm. Finally, Section 5 is the conclusion part.

## 2. Preliminaries

### 2.1. Fractional-Order Chaotic System

In the three-dimensional fractional chaotic system, the fractional differential operator is used instead of the standard differential as follows [13,14],

(1) $$\left\{ \begin{array}{l} \frac{d^{q}x}{dt^{q}}=ax+by\\ \frac{d^{q}y}{dt^{q}}=-bx+ay+c\sin(z)\\ \frac{d^{q}z}{dt^{q}}=dz+e\sin(x) \end{array}\right.$$

where *a*, *b*, *c*, *d* and *e* are system parameters, and *q* is the fractional order.

If the nonlinear terms sin(*x*) and sin(*z*) in the system (1) are replaced with cos(*x*) and cos(*z*), a new fractional-order chaotic system can be obtained.

(2) $$\left\{ \begin{array}{l} \frac{d^{q}x}{dt^{q}}=ax+by\\ \frac{d^{q}y}{dt^{q}}=-bx+ay+c\cos(z)\\ \frac{d^{q}z}{dt^{q}}=dz+e\cos(x) \end{array}\right.$$

**Figure 1:**
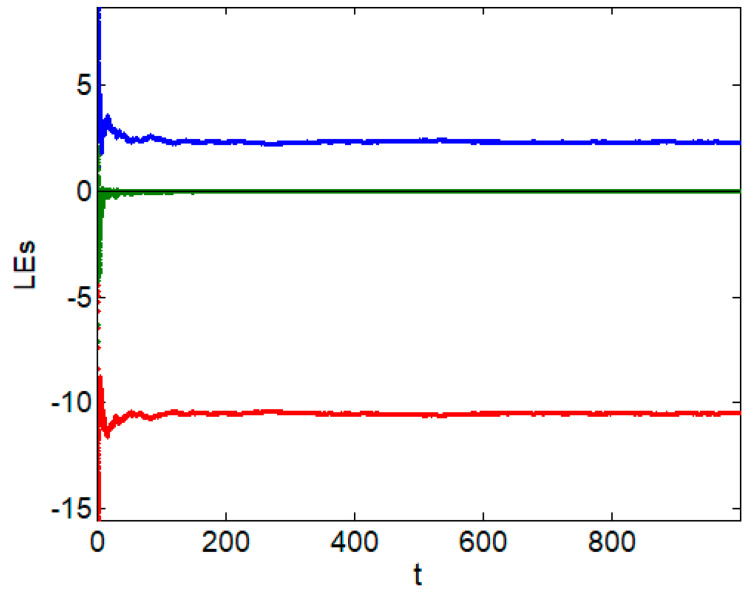
Lyapunov exponent.

**Figure 2:**
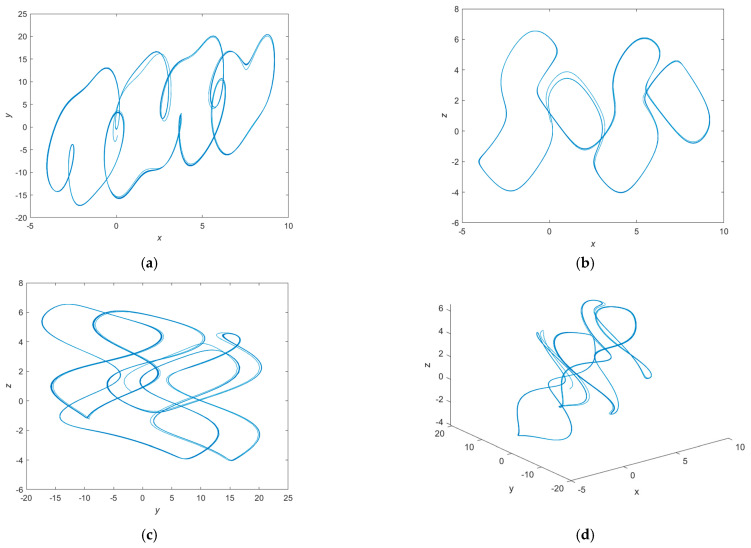
Fractional-order chaotic attractor. (**a**) *x*-*y* plane. (**b**) *x*-*z* plane. (**c**) *y*-*z* plane. (**d**) *x*-*y*-*z* plane.

In this paper, the fractional-order chaotic system parameters when *q* = 0.8, *a* = −1, *b* = 1, *c* = −80, *d* = −1 and *e* = 18 are selected. Meanwhile, the Lyapunov exponent of the system are 2.3111, −0.0037 and −10.4974, as shown in Figure 1, and the system exhibits chaotic behavior [15]. Figure 2 shows the chaotic attractors of the fractional-order chaotic system on different phase planes.

Different from integer-order chaotic systems, fractional-order chaotic systems have stronger memory characteristics and complex dynamic characteristics, can more accurately describe actual chaotic systems and are more in line with the actual needs of natural engineering applications. For image compression and encryption, it can generate more complex chaotic sequences and larger key spaces.

### 2.2. Compressed Sensing

Compressed sensing is a new signal sampling compression theory, which can realize image compression and encryption simultaneously during the sampling period [4,5]. Two-dimensional compressed sensing is to sample and measure the signal from two directions, which can not only further reduce the size of the compressed image, but also make the reconstructed image obtain better reconstruction quality. Assuming that the two-dimensional signal *x* is a signal of the size *N* × *N*, it can be sparsely expressed in specific domains such as DCT and DWT as,

(3) x=Ψs

where Ψ is the *N × N* orthogonal transform base matrix, and *s* is the sparse coefficient vector under Ψ. By linearly projecting *x* from two directions onto the measurement matrix Φ_1_ and Φ_2_ of size *M* × *N*, the *M* × *M* measurement value matrix *y* can be obtained.

(4) $$y=\Phi_{1}x\Phi_{2}^{T}=\Phi_{1}\Psi s\Phi_{2}^{T}=\Theta s$$

When the original signal *x* is to be reconstructed from the measured value *y*, the signal needs to be restored by solving the following convex optimization problem.

(5) $$\min{\left\|s\right\|}_{1}\;s.t.y=\Theta s$$

The existing reconstruction algorithms mainly include an orthogonal matching pursuit algorithm, a smooth l0 norm algorithm, a base pursuit algorithm, etc. This paper chooses a 2D projected gradient with an embedding decryption (2DPG-ED) algorithm [16] as the image reconstruction algorithm. Moreover, this paper will use the newly constructed measurement matrix to measure signals from two directions.

### 2.3. Multi-Parameter Fractional Quaternion Fourier Transform

Quaternion is a super-complex number with one real part and three imaginary parts [17]. In particular, when the quaternion modulus is 1, it is called a unit quaternion. When the real number term is 0, the quaternion is a pure quaternion. The quaternion function *f*(*x*,*y*) of multiple images can be expressed as [18],

(6) $$f(x,y)=f_{1}(x,y)+f_{2}(x,y)i+f_{3}(x,y)j+f_{4}(x,y)k$$

where *f*_1_(*x*,*y*), *f*_2_(*x*,*y*), *f*_3_(*x*,*y*) and *f*_4_(*x*,*y*) are represented as four color images in this paper.

A two-dimensional multi-parameter fractional quaternion Fourier transform model is defined as [12]:

(7) $$U_{M_{1},M_{2},\eta_{1},\eta_{2}}^{\alpha ,\beta ,\mu_{1},\mu_{2}}(f(x,y))=\sum_{u=0}^{M_{1}-1}\sum_{v=0}^{M_{2}-1}F_{u,v}^{\mu_{1},\mu_{2}}(f(x,y))\omega_{u}^{\alpha ,\mu_{1}}(\eta_{1})\omega_{v}^{\beta ,\mu_{2}}(\eta_{2})$$

where *u* and *v* are fractional orders, *μ*_1_ and *μ*_2_ are pure quaternions, *M*_1_ and *M*_2_ are arbitrary integers and *η*_1_ and *η*_2_ are real number vectors with the dimensions *M*_1_ and *M*_2_. $F_{u,v}^{\mu_{1},\mu_{2}}\left(f\left(x,y\right)\right)$ is a two-dimensional fractional quaternion Fourier transform and $\omega_{u}^{\alpha ,\mu_{1}}$ and $\omega_{v}^{\beta ,\mu_{2}}$ are weights. The coefficient is defined as follows:

(8) $$\omega_{u}^{\alpha ,\mu_{1}}(\eta )=\frac{1}{M_{1}}\sum_{k=0}^{M_{1}-1}\exp(-2\pi \mu_{1}/M_{1})[\alpha (k+\eta_{k}M_{1})-uk]$$

(9) $$\omega_{v}^{\beta ,\mu_{2}}(\eta )=\frac{1}{M_{2}}\sum_{k=0}^{M_{2}-1}\exp(-2\pi \mu_{2}/M_{2})[\beta (k+\eta_{k}M_{2})-vk]$$

The inverse transform of the multi-parameter fractional quaternion Fourier transform is $U_{M_{1},M_{2},\eta_{1},\eta_{2}}^{-\alpha ,-\beta ,\mu_{1},\mu_{2}}$.

## 3. Multiple-Image Compression and Encryption Algorithm Based on 2D CS and Optical Encryption

### 3.1. Fractional-Order Chaotic Sequence Generation

This paper calculates the initial values of the fractional-order chaotic system by calculating the SHA/MD5 hash value and external key *K* of four original images [19]. First, the 256-bit hexadecimal external key *K* is randomly generated, in 8-bit decimal format, which can be expressed as *K* = {*k*_1_, *k*_2_,…, *k*_32_}. Secondly, by XOR operation, the foreign key *K* is combined with the hash value H of the four images to obtain $K^{\prime }=\{k_{1}^{{}^{\prime }},\;k_{2}^{{}^{\prime }},\;\ldots ,\;k_{32}^{{}^{\prime }}\}$. Then, the initial value can be calculated according to Equation (10).

(10) $$\left\{ \begin{array}{l} x_{0}=\left(k_{1}^{{}^{\prime }}⊕k_{2}^{{}^{\prime }}⊕k_{3}^{{}^{\prime }}⊕k_{4}^{{}^{\prime }}⊕k_{5}^{{}^{\prime }}⊕k_{6}^{{}^{\prime }}\right)/256\\ y_{0}=\left(k_{7}^{{}^{\prime }}⊕k_{8}^{{}^{\prime }}⊕k_{9}^{{}^{\prime }}⊕k_{10}^{{}^{\prime }}⊕k_{11}^{{}^{\prime }}⊕k_{12}^{{}^{\prime }}\right)/256\\ z_{0}=\left(k_{13}^{{}^{\prime }}⊕k_{14}^{{}^{\prime }}⊕k_{15}^{{}^{\prime }}⊕k_{16}^{{}^{\prime }}⊕k_{17}^{{}^{\prime }}⊕k_{18}^{{}^{\prime }}\right)/256 \end{array}\right.$$

Finally, the initial value can be input into the new fractional-order chaotic equation to calculate the chaotic sequence.

### 3.2. Josephus Scrambling

Scrambling is an important step in encrypting images. This paper improves a Josephus scrambling algorithm [20], which makes it better for eliminating the correlation between image pixels. In the algorithm of this paper, the initial parameters are generated by the three-dimensional fractional-order chaotic sequence, which reduces the number of parameters that need to be passed, and the generated sequence is related to the number of cycles, which enhances the confusion of the sequence. The scrambling steps are as follows:

**Step 1**: Generate initial parameters. Assume that the size of the original image is *M* × *N*. Three groups of chaotic sequences are sequentially connected to form *S*, and the following four parameters are generated through the Formula (11),

(11) $$\left\{ \begin{array}{l} MI=1+sum(round(S(1:M)))\mathrm{mod}\;M\\ NI=1+sum(round(S(1:N)))\mathrm{mod}\;N\\ MIstep=sum(round(S(1:M)))\mathrm{mod}\;M\\ NIstep=sum(round(S(1:N)))\mathrm{mod}\;N \end{array}\right.$$

where *MI* and *NI* represent the position of the starting row and column, respectively, and *MIstep* and *NIstep* represent the number of steps moved by each row and column, respectively.

**Step 2:** The row sequence, *ros*, and column sequence, *cos*, are generated as follows: (1) Set vector *x* to 1 through *M* and vector *y* to 1 through *N*. (2) Initialize the first values of *ros* and *cos* to *MI* and *NI*, respectively, and then delete the *MI* and *NI* values in vector *x* and vector *y*. (3) Starting from the last deleted position, move the *MIstep* and *NIstep* steps in the circular vector *x* and *y*, and send the next value to the second position of *ros* and *cos*. (4) Update the values of *MIstep* and *NIstep* and add them to the number of rounds of this cycle, respectively. (5) Repeat the first two steps with rows *M* times and columns *N* times so that all values in vectors *x* and *y* have been offered to *ros* and *cos*.

**Step 3:** In the *i*-th row of the image, where *i* belongs to 1 and to *M*, the pixel points are sequentially replaced with pixel values {(*ros*(*cos*(1)) + *i*, *cos*(1))}, {(*ros*(*cos*(2)) + *i*, *cos*(2))}, …, {(*ros*(*cos*(*N*)) + *i*, *cos*(*N*))} in the following order. If the value of *ros*(*cos*(*k*)) + *i* (*k* = 1∼*N*) exceeds *M*, the value of *ros*(*cos*(*k*)) + *i* modulo *M* is taken as the replacement position instead of *ros*(*cos*(*k*)) + *i*.

**Step 4:** Repeat the previous step *M*-1 times. In each round, the *NI* value is updated with the value of *cos*(*N*), and a new *cos* column sequence is generated again in the way of step 2.

### 3.3. Measurement Matrix

In order to enhance the randomness of the chaotic sequence, discard the first 1000 values of each chaotic sequence and sample at equal intervals to ensure that the ratio of the values in the measurement matrix from the three sequences is one-third each. Simplify the total sequence to form a diagonal matrix *z_l_*, and then construct the total measurement matrix Φ as follows:

(12) $$\Phi =\left[\begin{array}{cccc} z_{l} & z_{l} & z_{l} & z_{l}\\ z_{l} & -z_{l} & z_{l} & -z_{l} \end{array}\cdots \begin{array}{cc} z_{l} & z_{l}\\ z_{l} & -z_{l} \end{array}\;\right]$$

Among them, the size of the diagonal matrix *z_l_* is *m*/2 × *m*/2. Extract *N* columns from matrix Φ to form a measurement matrix of size *m* × *N,* as shown in Algorithm 1.
**Algorithm 1** Generation of measurement matrixInput: chaotic sequence *x*, *y*, *z* compression ratio *m*/*N*Output: Measurement matrix *Phi*1: *X* = *x*(1001:*m*/2 + 1000);2: *Y* = *y*(1001:*m*/2 + 1000);3: *Z* = *z*(1001:*m*/2 + 1000);4: *W* = [*X Y Z*];5: *j* = 1:3:length(*W*);6: *Phi_t*1 = sqrt(2/*m*)*W(*j*);7: for *i* = 1:*m*/28:     if *Phi_t*1(*i*) < 09:       *Phi_t*2(*i*) = −1;10:   else11:     *Phi_t*2(*i*) = 1;12: end13: *Phi_t*3 = diag(*Phi_t2*);14: *Phi_t*4 = $\left[\begin{array}{ccccccc} Phi\_t3 & Phi\_t3 & Phi\_t3 & Phi\_t3 & \ldots & Phi\_t3 & Phi\_t3\\ Phi\_t3 & -Phi\_t3 & Phi\_t3 & -Phi\_t3 & \ldots & Phi\_t3 & -Phi\_t3 \end{array}\right]$;15: *Phi* = *Phi_t*4(1:*m*,1:*N*);

### 3.4. Image Compression and Encryption Algorithm

**Figure 3:**
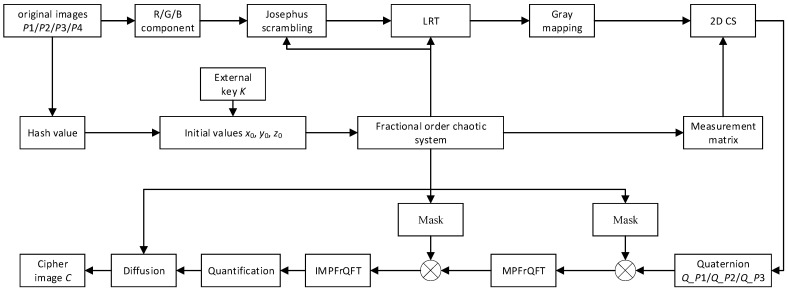
Multi-image compression and encryption flow chart.

The process of image encryption and compression is shown in Figure 3. It is mainly composed of two parts: (1) 2D CS is used to encrypt and compress four images. (2) In order to achieve better security performance, double random phase encoding based on MPFrQFT is used to further encrypt the compressed and encrypted image. Among them, the chaotic sequence of the fractional-order chaotic system is used in many places in the whole algorithm.

#### 3.4.1. Image Compression and Encryption Based on 2D CS

Assuming that the sizes of the four images *P*1, *P*2, *P*3 and *P*4 are all *N* × *N*, the compression steps are as follows:

**Step1:** According to Section 3.1, generate the initial values of the fractional-order chaotic equation and the chaotic sequences.

**Step2:** Extract the R, G and B components of the four images, respectively. According to Section 3.2, perform two-dimensional Josephus scrambling and linear random transformation (LRT) on the 12 components to obtain the encrypted image set *C*1.

The process of LRT transformation can be expressed as,

(13) $$C(i,j)=\left\{ \begin{array}{cc} C(i,j), & S(i,j)\mathrm{mod}\;2=0\\ P\_max-C(i,j) & S(i,j)\mathrm{mod}\;2=1 \end{array}\right.$$

where *C* is the encrypted image, *S* is the chaotic matrix generated by the fractional-order chaotic equation and *P_max* is the maximum pixel value of the encrypted image.

**Step3:** Generate the *M* × *N* measurement matrices Φ_1_ and Φ_2_ described in Section 3.3. Perform gray mapping on the image set *C*1 first to reduce the dynamic range of CS sampling and save the sampling bit width [16]. Then, according to formula (4), using the measurement matrices Φ_1_ and Φ_2_, the images are compressed and measured from two directions to obtain the compressed image set *C*2.

#### 3.4.2. Image Encryption with Double Random Phase Encoding Based on MPFrQFT

After completing the 2D CS, the paper further encrypts the image set *C*2 as follows.

**Step1:** Generate the quaternion *Q_P*1 according to formula (14) on the four compressed and encrypted images that were originally R components in the image set *C*2. In the same way, the G and B components can also generate quaternions *Q_P*2 and *Q_P*3.

(14) $$\left\{ \begin{array}{l} Q\_P1=R_{1}(x,y)+R_{2}(x,y)i+R_{3}(x,y)j+R_{4}(x,y)k\\ Q\_P2=G_{1}(x,y)+G_{2}(x,y)i+G_{3}(x,y)j+G_{4}(x,y)k\\ Q\_P3=B_{1}(x,y)+B_{2}(x,y)i+B_{3}(x,y)j+B_{4}(x,y)k \end{array}\right.$$

Here, *R_k_*, *G_k_* and *B_k_* (*k* = 1,2,3,4) are the R component, G component and B component of the four original images, respectively.

**Step2:** Right-multiply *Q_P*1, *Q_P*2 and *Q_P*3 by the quaternion random phase mask $e^{\mu_{1}2\pi a(x,y)}$ to obtain quaternion *Q_P*4, *Q_P*5 and *Q_P*6. Here, *μ*_1_ is a random unit pure quaternion, and the phase mask *a*(*x*,*y*) is composed of a chaotic sequence generated by a fractional-order chaotic system.

(15) $$\left\{ \begin{array}{l} Q\_P4=Q\_P1\times e^{\mu_{1}2\pi a(x,y)}\\ Q\_P5=Q\_P2\times e^{\mu_{1}2\pi a(x,y)}\\ Q\_P6=Q\_P3\times e^{\mu_{1}2\pi a(x,y)} \end{array}\right.$$

**Step3:** Perform MPFrQFT transformation on *Q_P*4, *Q_P*5 and *Q_P*6 to obtain *Q_P*7, *Q_P*8 and *Q_P*9, where *α*_1_ and *β*_1_ is the conversion level, *M*_1_ and *M*_2_ is the period, *η*_1_ and *η*_2_ is the random real vector in the *M*_1_ and *M*_2_ dimension and its values are independent and uniformly distributed in [0, 1000], *μ*_2_ is a random unit of the pure quaternion.

**Step4:** Right-multiply *Q_P*7, *Q_P*8 and *Q_P*9 by the quaternion random phase mask $e^{\mu_{3}2\pi b(u,v)}$ to obtain quaternion *Q_P*10, *Q_P*11 and *Q_P*12. Where *μ*_3_ is a random unit pure quaternion, and the phase mask *b*(*u*,*v*) is composed of a chaotic sequence generated by a fractional-order chaotic system.

(16) $$\left\{ \begin{array}{l} Q\_P10=Q\_P7\times e^{\mu_{3}2\pi b(u,v)}\\ Q\_P11=Q\_P8\times e^{\mu_{3}2\pi b(u,v)}\\ Q\_P12=Q\_P9\times e^{\mu_{3}2\pi b(u,v)} \end{array}\right.$$

**Step5:** Perform IMPFrQFT transform *Q_P*10, *Q_P*11 and *Q_P*12 to obtain *Q_P*13, *Q_P*14 and *Q_P*15, where *α*_2_ and *β*_2_ is the conversion level, *M*_3_ and *M*_4_ is the period, *η*_3_ and *η*_4_ is the random real vector in the *M*_3_ and *M*_4_ dimension, and its values are independent and uniformly distributed in [0, 1000], *μ*_4_ is a random unit of the pure quaternion.

**Step6:** Extract the real part and three imaginary parts of *Q_P*13, *Q_P*14 and *Q_P*15, respectively, and perform the quantization operation. The quantized images are arranged in order to form a 2*M* × 2*M* color image *C*3.

**Step7:** After performing a diffusion operation on each of the three layers of the color image *C*3, perform a diffusion operation on all the layers to obtain the final encrypted image *C*.

Among them, the diffusion method of each layer is as follows.

(17) $$C_{i}=\left\{ \begin{array}{ll} (P_{i}+P_{L}+P_{L-1}+floor(S_{i}\times 2^{F}))\;\mathrm{mod}\;F & \mathrm{if}\;i=1\\ (P_{i}+C_{i-1}+floor(S_{i}\times 2^{F}))\;\mathrm{mod}\;F & \mathrm{if}\;i\in [2,L] \end{array}\right.$$

where *P* is the image to be diffused, three chaotic sequences form the sequence *S_i_*, *L* is the total number of pixels in the image and *F* is the image depth.

### 3.5. Image Decryption and Reconstruction Algorithm

**Figure 4:**
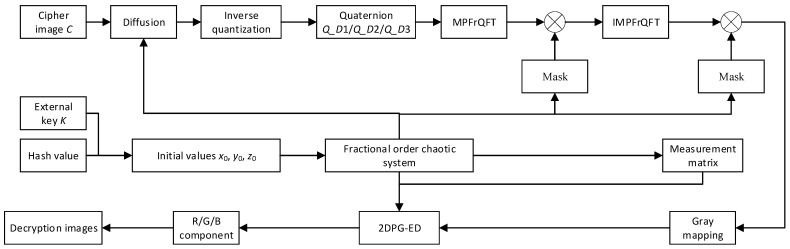
Multi-image decryption and reconstruction flowchart.

The decryption process is similar to the encryption process, as shown in Figure 4, which is the reverse operation of the encryption process. It is worth noting that the 2DPG-ED algorithm is used to reconstruct the image during the decryption process. The specific process is as follows.

#### 3.5.1. Image Decryption with Double Random Phase Encoding Based on MPFrQFT

**Step1:** According to the external key *K* and Hash value, generate the initial value of the fractional-order chaotic equation and the chaotic sequences.

**Step2:** Perform an inverse diffusion operation on each layer and all layers of the color image *C* to obtain the encrypted image *C*4.

**Step3:** Perform inverse quantization operation on all layers to get color image *C*5.

**Step4:** Generate the quaternion *Q_D*1 according to formula (18) on the four compressed and encrypted images that were originally R components in the image set *C*5. In the same way, the G component and the B component can also generate quaternions *Q_D*2 and *Q_D*3.

(18) $$\left\{ \begin{array}{l} Q\_D1=R_{1}(x,y)+R_{2}(x,y)i+R_{3}(x,y)j+R_{4}(x,y)k\\ Q\_D2=G_{1}(x,y)+G_{2}(x,y)i+G_{3}(x,y)j+G_{4}(x,y)k\\ Q\_D3=B_{1}(x,y)+B_{2}(x,y)i+B_{3}(x,y)j+B_{4}(x,y)k \end{array}\right.$$

where *R_k_*, *G_k_* and *B_k_* (*k* = 1, 2, 3, 4) are the R component, G component and B component of the four images to be decrypted, respectively.

**Step5:** Perform MPFrQFT transformation on *Q_D*1, *Q_D*2 and *Q_D*3 to obtain *Q_D*4, *Q_D*5 and *Q_D*6, where the parameters are *α*_2_, *β*_2_, *M*_3_, *M*_4_, *η*_3_, *η*_4_ and *μ*_4_.

**Step6:** Right-multiply *Q_D*4, *Q_D*5 and *Q_D*6 by the quaternion random phase mask $e^{-\mu_{3}2\pi b(u,v)}$ to obtain quaternion *Q_D*7, *Q_D*8 and *Q_D*9.

(19) $$\left\{ \begin{array}{l} Q\_D7=Q\_D4\times e^{-\mu_{3}2\pi b(u,v)}\\ Q\_D8=Q\_D5\times e^{-\mu_{3}2\pi b(u,v)}\\ Q\_D9=Q\_D6\times e^{-\mu_{3}2\pi b(u,v)} \end{array}\right.$$

**Step7:** Perform IMPFrQFT transformation on *Q_D*7, *Q_D*8 and *Q_D*9 to obtain *Q_D*10, *Q_D*11 and *Q_D*12, where the parameters are *α*_1_, *β*_1_, *M*_1_, *M*_2_, *η*_1_, *η*_2_ and *μ*_2_.

**Step8:** Right-multiply *Q_D*10, *Q_D*11 and *Q_D*12 by the quaternion random phase mask $e^{-\mu_{1}2\pi a(x,y)}$ to obtain quaternion *Q_D*13, *Q_D*14 and *Q_D*15.

(20) $$\left\{ \begin{array}{l} Q\_D13=Q\_D10\times e^{-\mu_{1}2\pi a(x,y)}\\ Q\_D14=Q\_D11\times e^{-\mu_{1}2\pi a(x,y)}\\ Q\_D15=Q\_D12\times e^{-\mu_{1}2\pi a(x,y)} \end{array}\right.$$

#### 3.5.2. 2D CS Image Reconstruction

**Step1:** Extract the real part and three imaginary parts in *Q_D*13, *Q_D*14 and *Q_D*15, respectively, and arrange them in order to form a 2*M* × 2*M* color image *C*6.

**Step2:** Generate measurement matrices Φ_1_ and Φ_2_ again.

**Step3:** First, divide the R, G and B layers of color image *C*6 into 12 layers by the size of the original image, then perform grayscale mapping, and then reconstruct the image according to the 2DPG-ED algorithm. The reconstruction algorithm includes the inverse operation of two-dimensional Josephus scrambling and LRT transformation.

**Step4:** Turn the reconstructed 12 layers back to the original four images according to the original encryption order of the image.

## 4. Simulation Results and Analysis

In order to verify the feasibility of the proposed compression and encryption algorithm, a series of numerical simulations were carried out on a computer equipped with CPU @ 2.10 GHz, 16G RAM and MATLAB R2019b. The initial parameters of the fractional-order chaotic system are set to *x*_0_ = 0.1, *y*_0_ = 0.1 and *z*_0_ = 0.1. The compression ratio (CR) is 0.5. The parameters of the double random phase encoding part are set as: period *M*_1_ = 23, *M*_2_ = 29, *M*_3_ = 25 and *M*_4_ = 27, the vector parameter *η_s_* (*s* = 1,2,3,4) are *Ms*-dimensional random arbitrary real vectors and the unit pure quaternion arrays *µ*_1_, *µ*_2_, *µ*_3_ and *µ*_4_ are *µ*_1_ = (*i* + *j* + *k*)/3, *µ*_2_ = *i*, *µ*_3_ = *j* and *µ*_4_ = *k*. Set *α*_1_ = 3.8287, *α*_2_ = 3.2011, *β*_1_ = 1.9415 and *β*_2_ = 0.5675. Furthermore, the algorithm is also suitable for the multi-image compression and encryption of grayscale images. This paper realizes double images compression and encryption by setting two imaginary parts equal to zero, and the decrypted image is obtained by the corresponding non-zero imaginary part.

### 4.1. Experimental Results

**Figure 5:**
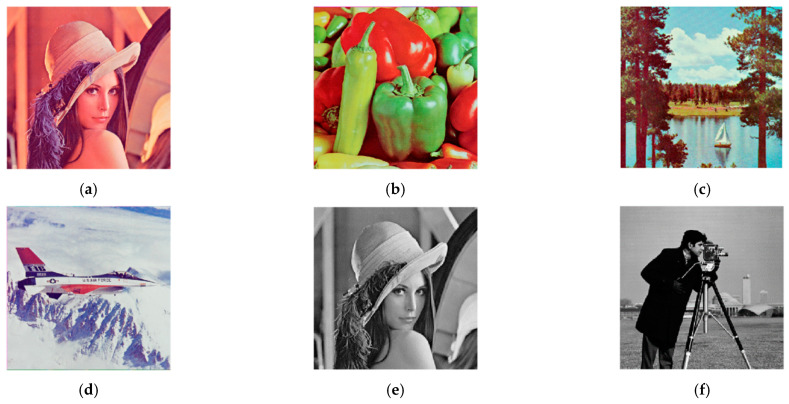
Original image. (**a**) Lena512. (**b**) Peppers512. (**c**) Lake512. (**d**) Airplane512. (**e**) Lena256. (**f**) Cameraman256.

**Figure 6:**
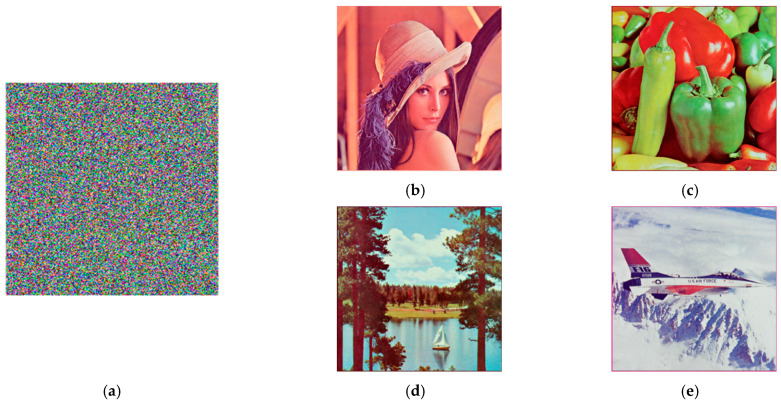
Color images’ encryption and decryption results. (**a**) Encrypted image of 4 color images. (**b**) Decrypted Lena512 image. (**c**) Decrypted Peppers512 image. (**d**) Decrypted Lake512 image. (**e**) Decrypted Airplane512 image.

**Figure 7:**
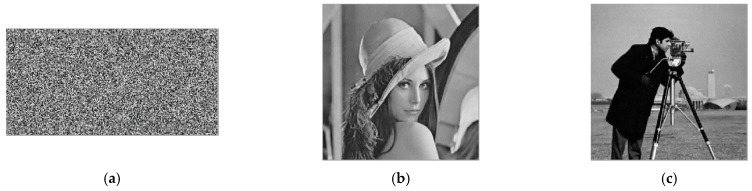
Grayscale images’ encryption and decryption results. (**a**) Encrypted image of 2 grayscale images. (**b**) Decrypted Lena256 image. (**c**) Decrypted Cameraman256 image.

The test images include 512 × 512 color images: Lena, Peppers, Lake, Airplane, and 256 × 256 grayscale images: Lena, Cameraman. To distinguish the images, the specific image is indicated by the combination of the image name and the image size, as shown in Figure 5. The last three of the color images are from the USC-SIPI image library, while Lena and Cameraman are commonly used images. The image encryption and decryption results are shown in Figure 6 and Figure 7.

It can be seen from Figure 6 and Figure 7 that both color images and grayscale images can be effectively compressed and encrypted by the algorithm in this paper, and the decrypted image is visually very similar to the original image.

### 4.2. Compression Performance Analysis

In this paper, 2D CS is used to compress and encrypt multiple images simultaneously. The encrypted image is compressed into different sizes according to CR. The larger the CR, the better the reconstruction quality of the decrypted image. Both the peak signal-to-noise ratio (PSNR) and average structural similarity (MSSIM) are used to evaluate the compression performance of the algorithm. PSNR is a common indicator for appraising the quality of decrypted images. The calculation method is as follows:

(21) $$\mathrm{PSNR}=10\log\frac{255^{2}}{\left(1/3MN\right)\sum_{i=1}^{M}\sum_{i=1}^{N}{\left[P\left(i,j\right)-D\left(i,j\right)\right]}^{2}}$$

where *M* and *N* are the sizes of the image, and *P*(*i*,*j*) and *D*(*i*,*j*) are the pixel values at row *i* and column *j* in the original image and the decrypted image, respectively. The larger the PSNR, the smaller the image distortion.

MSSIM is an indicator of similarity, and its distribution range is between 0 and 1. The larger the test value, the stronger the similarity between images. It is defined as:

(22) $$\mathrm{SSIM}(x,y)=\frac{\left(2\mu_{x}\mu_{y}+C_{1})(2\sigma_{xy}+C_{2}\right)}{\left(\mu_{x}^{2}+\mu_{y}^{2}+C_{1})(\sigma_{x}^{2}+\sigma_{y}^{2}+C_{2}\right)}$$

(23) $$\mathrm{MSSIM}=\frac{1}{N}\sum_{j=1}^{N}\mathrm{SSIM}(x_{j},y_{j})$$

where *C*_1_ = (*k*_1_*L*)^2^, *C*_2_ = (*k*_2_*L*)^2^, *k*_1_ = 0.01, *k*_2_ = 0.03 and *L =* 255. *µ_x_*, *µ_y_*, *σ_x_*, *σ_y_* and *σ_xy_* represent the mean, variance and covariance of the original image and the decrypted image, respectively. *x_j_* and *y_j_* represent the two images of the *j*-th window and *N* is the total number of windows, where *N* = 64. Table 1 shows the PSNR and MSSIM of the decryption results of the algorithm in this paper under different CRs.

From Table 1, we can see that both the PSNR and MSSIM of the decrypted image are in a range with better results. As the CR value increases, the value of MSSIM also increases. The higher the similarity between the decrypted image and the original image, the better the image reconstruction effect. The results in Table 1 show that this algorithm can compress and encrypt images in a diversified manner.

Table 2 shows the PSNR results of different images under different compression ratios, as well as comparisons with references. From Table 2, we can see that our reconstruction effect is significantly better than the reference [7,21,22].

### 4.3. Statistical Analysis

#### 4.3.1. Histogram

**Figure 8:**
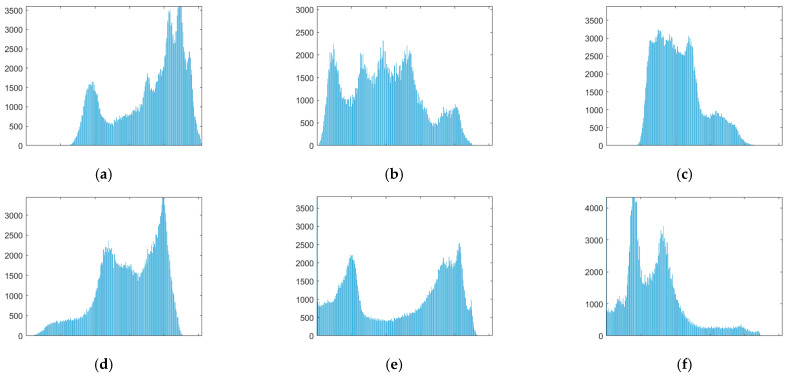

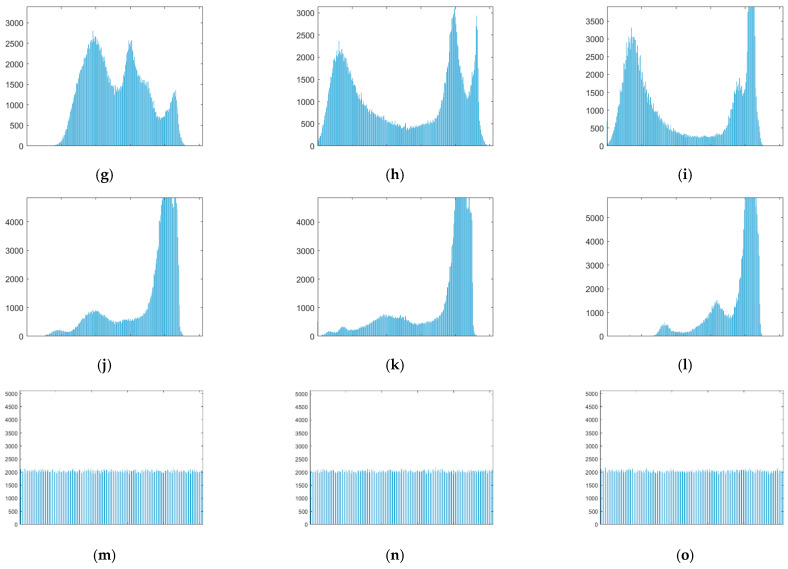
Histograms of color image and ciphertext. (**a**) Histogram of Lena512 R. (**b**) Histogram of Lena512 G. (**c**) Histogram of Lena512 B. (**d**) Histogram of Peppers512 R. (**e**) Histogram of Peppers512 G. (**f**) Histogram of Peppers512 B. (**g**) Histogram of Lake512 R. (**h**) Histogram of Lake512 G. (**i**) Histogram of Lake512 B. (**j**) Histogram of Airplane512 R. (**k**) Histogram of Airplane512 G. (**l**) Histogram of Airplane512 B. (**m**) Histogram of encrypted image R. (**n**) Histogram of encrypted image G. (**o**) Histogram of encrypted image B.

**Figure 9:**
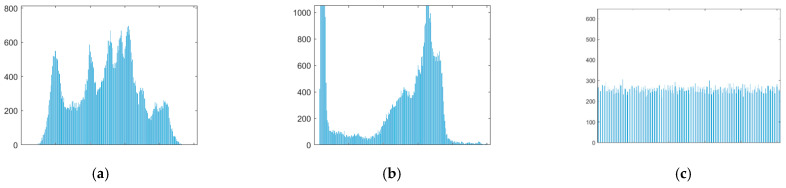
Histograms of grayscale image and ciphertext. (**a**) Histogram of Lena256. (**b**) Histogram of Cameraman256. (**c**) Histogram of encrypted image.

The histogram reflects the relationship between the frequency of each gray level pixel in the image and the gray level, and it is one of the important criteria for evaluating the security performance of an image encryption scheme. If the histograms of encrypted images are relatively evenly distributed, the encryption effect is good. Figure 8 is the histogram of the respective red, green and blue components of the color images and the histogram of the total encrypted text image of the four images. Figure 9 is the histogram of the two grayscale images and the total cipher text image.

From Figure 8 and Figure 9, it can be found that the histograms of the color ciphertext image and the grayscale ciphertext image are very uniform. This means that any statistical information of plaintext cannot be obtained from the histogram of the ciphertext. This shows that the multi-image compression–encryption algorithm can effectively resist statistical analysis attacks.

#### 4.3.2. Correlation between Adjacent Pixels

**Figure 10:**
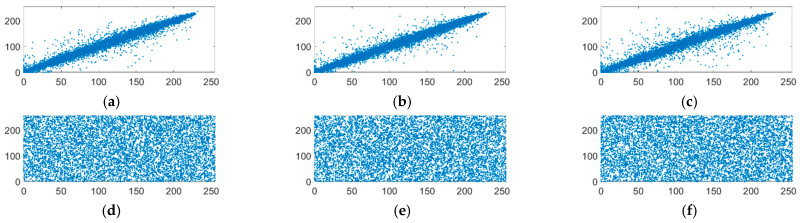
The relative distribution of the original image “Peppers512” and the encrypted image in three directions. (**a**) Correlation distribution in the horizontal direction of the Peppers512. (**b**) Correlation distribution in the vertical direction of the Peppers512. (**c**) Correlation distribution in the diagonal direction of the Peppers512. (**d**) Correlation distribution in the horizontal direction of the ciphertext image. (**e**) Correlation distribution in the vertical direction of the ciphertext image. (**f**) Correlation distribution in the diagonal direction of the ciphertext image.

The correlation between adjacent pixels can also be used to obtain information about the original image. Strongly correlated pixels exist in the original image, and image encryption can destroy this correlation. The correlation coefficient *ρ_xy_* is defined as follows:

(24) $$\rho_{xy}=\frac{E((x-E(x))(y-E(y)))}{\sqrt{D(x)D(y)}}$$

where *E*(*x*) and *D*(*x*) are the mean and variance of x, respectively, and the same is true for *E*(*y*) and *D*(*y*). The value of *ρ_xy_* is in the range of 0 to 1, and the closer to 1, the stronger the correlation. Table 3 shows the correlation coefficients between the proposed multi-image compression–encryption algorithm and references. Figure 10 shows the relative distribution of adjacent pixels of the “Peppers512” image and its ciphertext image.

It can be seen from Table 3 that the correlation coefficients of the ciphertext image of the color image and the grayscale image are close to 0, and are basically better than the values in references [23,24,25], indicating that our encryption method can resist statistics attacks and have higher security.

Figure 10 shows the pixel distribution of “Peppers512” in different directions. Compared to the diagonal pixel distribution of the original image, the ciphertext image is uniformly distributed in the entire coordinate space.

#### 4.3.3. Information Entropy

Information entropy is used to evaluate the randomness and unpredictability of the image, which is calculated as follows:

(25) $$H(m)=\sum_{i=1}^{N}p(m_{i})\log_{2}\frac{1}{p(m_{i})}$$

where *p*(*m_i_*) is the probability of the occurrence of image pixel gray value *m_i_*, and *N* is the total number of *m_i_*. Among the values of information entropy, eight is an ideal value. The information entropy values of color images, grayscale images and their encrypted images are shown in Table 4 and Table 5.

Obviously, it can be seen from Table 4 and Table 5 that the information entropy of the R component, G component and B component of the color ciphertext image exceeds 7.99, which is better than the information entropy of the reference [26]. The information entropy of the grayscale ciphertext image is also around eight. This means that the image encrypted by this algorithm has good randomness.

Local Shannon entropy is an index that quantitatively describes the randomness of an image from a local perspective, which can be written as,

(26) $$\overset{\_\_\_\_\_\_}{H_{k,T_{B}}}(S)=\sum_{i=1}^{k}\frac{H(S_{i})}{k}$$

where *S_1_∼S_k_* are non-overlapping image blocks, *k* is the number of selected blocks, *T_B_* represents the number of pixels in each selected image block and *H*(*S_i_*) is the information entropy of the selected image block. If the value of local Shannon entropy is in the interval ($h_{left}^{l*},h_{right}^{l*}$), the image will pass the local Shannon entropy test. In this paper, *k* and *T_B_* are selected as 30 and 1936. Table 6 shows the local Shannon entropy of the color ciphertext image.

According to Table 6, it can be known that the color ciphertext images have passed the test, and the local Shannon entropy of the total ciphertext image is also within the range of values. This means that the ciphertext image of the algorithm in the paper has good randomness.

### 4.4. Key Space Analysis

To resist brute force attacks, the key space of a secure image compression–encryption algorithm is considered to be at least 2^100^. In this algorithm, the key is mainly composed of the following two parts: the 256-bit initial key K and the 256-bit hash value of the original image. Multiple parameters in MPFrQFT can also be used as a key during the transfer process. By simple addition, it can be seen that the key space of the algorithm is at least 2^512^, which is greater than 2^100^. Table 7 gives the comparison results of key space with other algorithms.

As can be seen in Table 7, the proposed scheme has the largest key space than other encryption schemes. So the algorithm can meet the security requirements of the key space and resist brute force attacks.

### 4.5. Key Sensitivity Analysis

Key sensitivity requires that the encryption algorithm can produce completely different encryption results due to the slight change of key. This means that only a unique and correct key can recover the plaintext. In this paper, two keys, *K_a_* and *K_b_*, are randomly selected during verification, but only 1-bit difference is guaranteed between the two keys.

*K_a_* = ‘4ffDc1EB6bFfC9ac5Abe63a7BBe20c6EF6BDdDEcAc09eb8ECC8dDEe4e18eEBaA’

*K_b_* = ‘4ffDc1EB6bFfC9ac5Abe63a7BBe20c6EF6BDdDEcAc09eb8ECC8dDEe4e18eEBaB’

**Figure 11:**
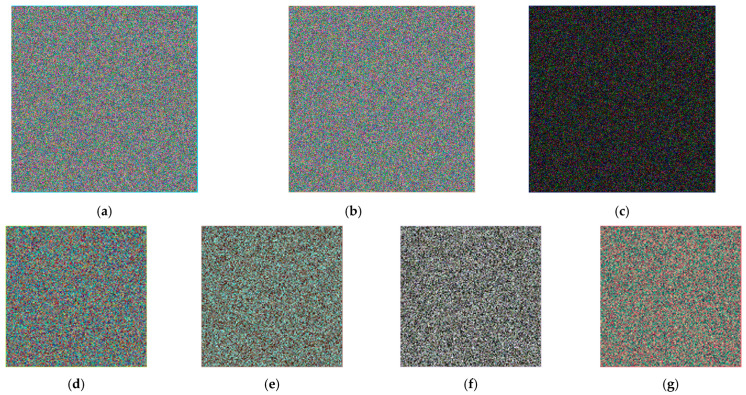
Key sensitivity analysis. (**a**) Image *C_a_* encrypted with *K_a_*. (**b**) Image *C_b_* encrypted with *K_b_*_._ (**c**) Differences between encrypted images. (**d**) Use *K_b_* to decrypt the Lena512 after ciphertext *C_a_*. (**e**) Use *K_b_* to decrypt the Peppers512 after ciphertext *C_a_*. (**f**) Use *K_b_* to decrypt the Lake512 after ciphertext *C_a_*. (**g**) Use *K_b_* to decrypt the Airplane512 after ciphertext *C_a_*.

In the simulation, the results of encrypted and decrypted four color images using keys *K_a_* and *K_b_* are shown in Figure 11.

From Figure 11a–c, we can see that with the original image encrypted with *K_a_* and *K_b_*, two visually secure encrypted results can be obtained, and the difference between them is larger. Moreover, as shown in Figure 11d–g, using the key that is 1-bit different from the correct key for decryption, none of the four original images can be reconstructed correctly. Therefore, the proposed multi-image compression–encryption algorithm is key sensitive.

### 4.6. Differential Attack Analysis

The differential attack is an effective and common attack against security. After adding slight changes to the original image, the attacker can obtain usable information by analyzing the differences between the two encrypted images. The pixel change rate (NPCR), uniform average change intensity (UACI) and avalanche effect are three common indicators to evaluate the impact of differential attacks. If the plaintext pixel value changes slightly, the ciphertext pixel value can change significantly after encryption, which shows the algorithm has good randomization characteristics. The calculation methods of NPCR and UACI are as follows:

(27) $$\mathrm{NPCR}=\frac{\sum_{i,j}D\left(i,j\right)}{W\times H}\times 100\%$$

(28) $$\mathrm{UACI}=\frac{1}{W\times H}\left[\sum_{i,j}\frac{\left|d_{1}\left(i,j\right)-d_{2}\left(i,j\right)\right|}{255}\right]\times 100\%$$

where *W* and *H* represent the width and height of the image, respectively, and *d*_1_ and *d*_2_ are the two ciphertext images before and after the plaintext image changes one-pixel value. If *d*_1_(*i*,*j*) = *d*_2_(*i*,*j*), then *D*(*i*,*j*) = 0, otherwise, *D*(*i*,*j*) = 1. The ideal expected values of the NPCR, UACI and avalanche effects are 99.6094%, 33.4635% and 50%, respectively. Table 8 shows the NPCR, UACI and avalanche effect values, as well as the comparison results with other algorithms.

The results in Table 8 show that the values of the NPCR, UACI and avalanche effect of ciphertext images are close to their theoretical values and are better than the algorithms in reference [25]. Therefore, this algorithm has a good performance of resistance to differential attacks.

### 4.7. Robustness Analysis

#### 4.7.1. Analysis of Noise Attacks

**Figure 12:**
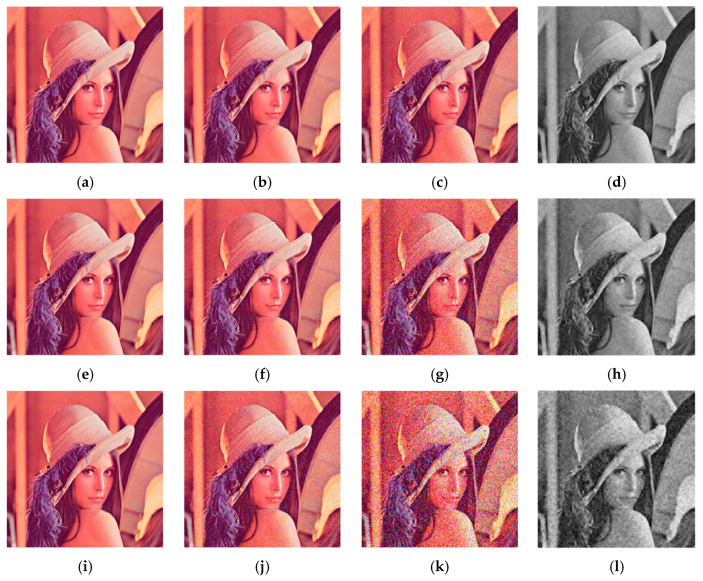
Noise attack results with different noise intensities. (**a**) 10^−5^ SPN. (**b**) 10^−4^ SPN. (**c**) 10^−3^ SPN. (**d**) 10^−3^ SPN. (**e**) 10^−6^ SN. (**f**) 10^−4^ SN. (**g**) 10^−3^ SN. (**h**) 10^−3^ SN. (**i**) 10^−6^ GN. (**j**) 10^−4^ GN. (**k**) 10^−3^ GN. (**l**) 10^−3^ GN.

During the transmission process, the ciphertext image will inevitably be affected by noise and interference. To evaluate the impact of noise and interference on the decrypted image, salt and pepper noise (SPN), speckle noise (SN) and Gaussian noise (GN) were added to the encrypted image to measure the ability of the algorithm to resist noise attacks. Table 9 shows the decrypted results after adding different types and degrees of noise to the ciphertext image, as well as the comparison with other algorithms. Figure 12 is a decrypted image of a noise image.

Table 9 shows that our algorithm can resist 10^−5^ SPN, 10^−6^ GN and SN. Compared with reference [23], the anti-noise effect of our algorithm has obvious advantages when the noise is greater.

Figure 12 shows the effect of decrypting images under higher levels of noise. It can be found that when the noise is 10^−3^ GN and SN, there are obvious spots in the decrypted image, but the original image can still be easily recognized. When the noise is 10^−3^ SPN, the quality of the decrypted image is still good. It shows the algorithm can resist at least 10^−3^ noise attacks, and the ability to resist salt and pepper noise is stronger than that of other types of noise.

#### 4.7.2. Analysis of Shear Attack

**Figure 13:**
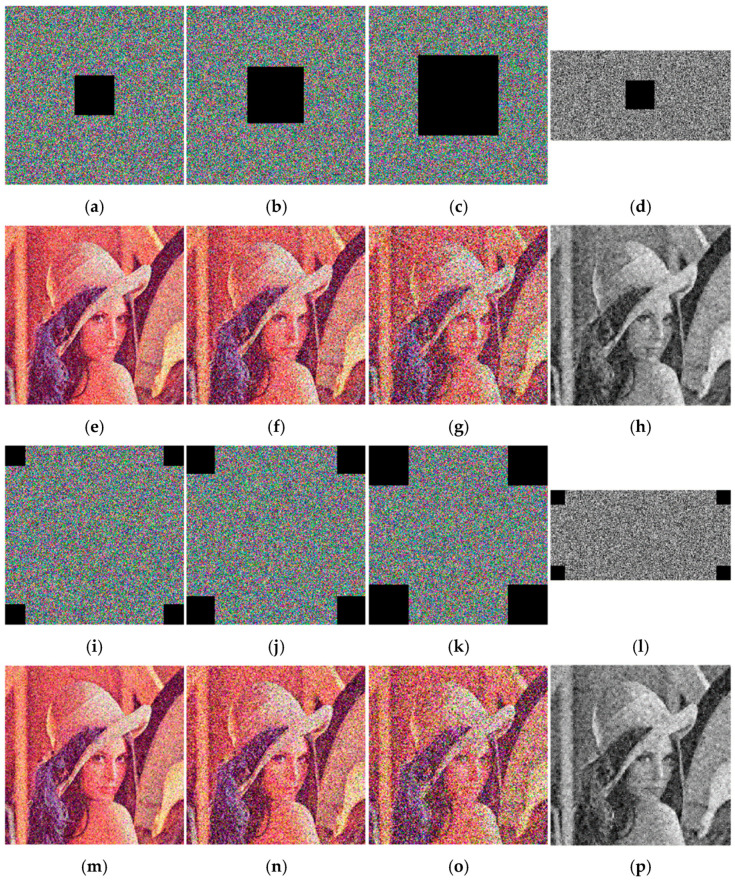
Results of shearing attacks of different strengths. (**a**) 5% data loss. (**b**) 10% data loss. (**c**) 20% data loss. (**d**) 5% data loss. (**e**) decrypted image of (**a**). (**f**) decrypted image of (**b**). (**g**) decrypted image of (**c**). (**h**) decrypted image of (**d**). (**i**) 5% data loss. (**j**) 10% data loss. (**k**) 20% data loss. (**l**) 5% data loss. (**m**) decrypted image of (**i**). (**n**) decrypted image of (**j**). (**o**) decrypted image of (**k**). (**p**) decrypted image of (**l**).

The ciphertext may also be subject to clipping attacks during transmission, so the compression and encryption algorithm needs to have the ability to resist clipping attacks. Figure 13 shows the decrypted image recovery result after the ciphertext image has been subjected to different cutting methods and varying degrees of data loss.

Figure 13 shows that the main information of the original image can still be roughly represented after being subjected to different degrees of shearing attacks. As the amount of data loss increases, the recovery effect is gradually declining. As far as this algorithm is concerned, the cutting position has little effect on the image restoration effect. All in all, our algorithm can resist shearing attacks to a certain degree.

## 5. Conclusions

This paper proposes a multi-image encryption and compression algorithm based on 2D CS and optical encryption. This paper first uses 2D CS to compress and encrypt multiple images simultaneously, and then uses MPFrQFT-based double random phase encoding to encrypt the images twice, which enhances the encryption effect. Among them, a newly designed structured measurement matrix is used in 2D CS, which can effectively reduce the transmission load, and so the encryption of multiple images is realized in MPFrQFT. In addition, the chaotic sequence generated by the fractional chaotic system is used in the sampling process of 2D CS and the double random phase encoding process. This algorithm combines the advantages of compression–encryption and multi-image encryption, which can further reduce the amount of data transmission and key transmission consumption, while increasing the encryption capacity and ensuring the security of the image. Experimental results show that this compression–encryption algorithm has good robustness and compression performance, can resist at least 10^−3^ noise attacks and shear attacks with 20% data loss and can obtain better recovery results under different compression ratios, which is better than the current reference data. In terms of security performance, it has 2^512^ key space and can resist a statistical analysis attack and differential attack.

## Figures and Tables

**Table 1:** PSNR and MSSIM of plaintext image and decrypted image.

Images	CR	PSNR	MSSIM
Lena512	0.75	40.0126	0.9929
	0.5	36.5825	0.9844
	0.25	33.6496	0.9704
Peppers512	0.75	38.4215	0.9909
	0.5	35.1721	0.9806
	0.25	32.7130	0.9671
Lake512	0.75	34.9974	0.9776
	0.5	31.6709	0.9593
	0.25	28.8113	0.9317
Airplane512	0.75	42.0901	0.9832
	0.5	38.6556	0.9662
	0.25	34.9643	0.9371
Lena256	0.75	41.0852	0.9981
	0.5	36.7034	0.9947
	0.25	32.2037	0.9850
Cameraman256	0.75	38.8366	0.9966
	0.5	34.0923	0.9904
	0.25	30.0109	0.9737

**Table 2:** Compression performance of different algorithms.

Images	CR	PSNR	Ref. [21]	Ref. [22]	Ref. [7]
Peppers512	0.75	38.4215	31.7121	-	<30
	0.5	35.1721	26.4965	-	<30
	0.25	32.7130	14.0119	-	>25
Lena256	0.75	41.0852	-	36.1415	-
	0.5	36.7034	-	32.1471	-
	0.25	32.2037	-	28.0615	-

**Table 3:** The adjacent pixel correlation coefficient of original image and ciphertext image.

Images		Horizonta	Vertical	Diagonal
Lena512	Plain image	0.9881	0.9775	0.9698
	Cipher image	−0.0014	−0.0013	−0.0030
	Ref. [23]	0.0019	0.0184	−0.0129
Peppers512	Plain image	0.9822	0.9786	0.9693
	Cipher image	0.0017	-0.0008	−0.0014
	Ref. [24]	0.0004	−0.0002	0.0019
Lake512	Plain image	0.9879	0.9774	0.9696
	Cipher image	−0.0018	−0.0009	−0.0013
	Ref. [23]	0.0047	−0.0021	−0.0093
Airplane512	Plain image	0.9588	0.9586	0.9246
	Cipher image	−0.0016	0.0003	−0.0006
	Ref. [24]	−0.0008	−0.0042	−0.0030
Lena256	Plain image	0.9703	0.9413	0.9152
	Cipher image	0.0037	−0.0024	0.0038
	Ref. [25]	−0.0018	−0.0070	−0.0024
Cameraman256	Plain image	0.9595	0.9334	0.9084
	Cipher image	0.0082	−0.0011	−0.0004
	Ref. [25]	−0.0096	0.0082	−0.0043

**Table 4:** Information entropy of color images and ciphertext image.

Images		R Component	G Component	B Component
Lena512	Plain image	7.2531	7.5940	6.9684
	Cipher image	7.9987	7.9985	7.9987
	Ref. [26]	7.9970	7.9972	7.9967
Peppers512	Plain image	7.3388	7.4963	7.0583
	Cipher image	7.9985	7.9987	7.9986
	Ref. [26]	7.9974	7.9972	7.9971
Lake512	Plain image	7.3124	7.6429	7.2136
	Cipher image	7.9986	7.9986	7.9987
	Ref. [26]	7.9970	7.9967	7.9969
Airplane512	Plain image	6.7178	6.7990	6.2138
	Cipher image	7.9988	7.9986	7.9986
	Ref. [26]	7.9974	7.9972	7.9980

**Table 5:** Information entropy of grayscale image and ciphertext image.

Images	Plain Image	Cipher Image
Lena256	7.4443	7.9952
Cameraman256	7.0097	7.9939

**Table 6:** The local Shannon entropy of the color ciphertext image.

Image	Lena512	Peppers512	Lake512	Airplane512	Mean
Local Shannon entropy	7.901517	7.903376	7.901792	7.901701	7.902096
$h_{left}^{l*}/h_{right}^{l*}$ = 7.901515698/7.903422936					

**Table 7:** Comparison of key space of the proposed method with other methods.

Algorithm	Ours	Ref. [25]	Ref. [27]	Ref. [28]	Ref. [7]
Key Space	>2^512^	≈2^372^	≈2^210^	≈2^233^	≈2^294^

**Table 8:** NPCR, UACI and avalanche effect values of ciphertext images.

Images	Algorithm	NPCR	UACI	Avalanche Effect
Lena512	Ours	99.6078	33.4741	50.0094
	Ref. [29]	99.7470	36.7368	-
Peppers512	Ours	99.6088	33.4719	50.0022
	Ref. [29]	99.7509	34.7577	-
Lake512	Ours	99.6080	33.4716	49.9982
	Ref. [29]	-	-	-
Airplane512	Ours	99.6106	33.4777	50.0072
	Ref. [29]	99.7548	46.6056	-
Lena256	Ours	99.6087	33.4924	50.0151
	Ref. [22]	99.6101	33.4654	-
Cameraman256	Ours	99.6075	33.4671	49.9867
	Ref. [22]	99.6066	33.4679	-

**Table 9:** PSNR of the decrypted image of the noisy ciphertext image.

Noise Type	Noise Intensity	Lena512 Ours	Lena512 [23]	Peppers512	Lake512	Airplane512
SPN	1 × 10^−5^	36.2751	32.2248	34.9380	31.5531	38.1682
	3 × 10^−5^	35.9222	31.8791	34.6932	31.3934	37.6614
	5 × 10^−5^	35.4256	29.6256	34.3170	31.1725	36.9469
SN	1 × 10^−6^	36.5825	32.6759	35.1716	31.6857	38.6497
	3 × 10^−6^	35.9578	17.2882	34.7033	31.4104	37.6926
	5 × 10^−6^	35.0968	13.5636	34.0699	31.0119	36.4843
GN	1 × 10^−6^	35.9662	18.0331	34.7121	31.3983	37.7041
	3 × 10^−6^	34.4031	13.1115	33.5063	30.6553	35.4992
	5 × 10^−6^	33.5791	9.2767	32.8993	30.2160	34.4459
